# Non-Heme-Binding Domains and Segments of the *Staphylococcus aureus* IsdB Protein Critically Contribute to the Kinetics and Equilibrium of Heme Acquisition from Methemoglobin

**DOI:** 10.1371/journal.pone.0100744

**Published:** 2014-06-24

**Authors:** Hui Zhu, Dengfeng Li, Mengyao Liu, Valerie Copié, Benfang Lei

**Affiliations:** 1 Department of Microbiology and Immunology, Montana State University, Bozeman, Montana, United States of America; 2 Department of Chemistry and Biochemistry, Montana State University, Bozeman, Montana, United States of America; 3 Department of Physiology, Harbin Medical University, Harbin, People's Republic of China; University of Texas-Huston Medical School, United States of America

## Abstract

The hemoglobin receptor IsdB rapidly acquires heme from methemoglobin (metHb) in the heme acquisition pathway of *Staphylococcus aureus*. IsdB consists of N-terminal segment (NS), NEAT1 (N1), middle (MD), and heme binding NEAT2 (N2) domains, and C-terminal segment (CS). This study aims to elucidate the roles of these domains or segments in the metHb/IsdB reaction. Deletion of CS does not alter the kinetics and equilibrium of the reaction. Sequential deletions of NS and N1 in NS-N1-MD-N2 progressively reduce heme transfer rates and change the kinetic pattern from one to two phases, but have no effect on the equilibrium of the heme transfer reaction, whereas further deletion of MD reduces the percentage of transferred metHb heme. MD-N2 has higher affinity for heme than N2. MD *in trans* reduces rates of heme dissociation from holo-N2 and increases the percentage of metHb heme captured by N2 by 4.5 fold. NS-N1-MD and N2, but not NS-N1, MD, and N2, reconstitute the rapid metHb/IsdB reaction. NS-N1-MD-N^IsdC^, a fusion protein of NS-N1-MD and the NEAT domain of IsdC, slowly acquires heme from metHb by itself but together with N2 results in rapid heme loss from metHb. Thus, NS-N1 and MD domains specifically and critically contribute to the kinetics and equilibrium of the metHb/IsdB reaction, respectively. These findings support a mechanism of direct heme acquisition by IsdB in which MD enhances the affinity of N2 for heme to thermodynamically drive heme transfer from metHb to IsdB and in which NS is required for the rapid and single phase kinetics of the metHb/IsdB reaction.

## Introduction

Iron is essential for growth and survival of most bacteria. There is little free iron to support bacterial growth in mammalian hosts, which is due to the extremely low solubility of ferric iron in water at physiological pH and the presence of proteins that avidly bind iron. Furthermore, most iron in mammals is sequestered as a complex with protoporphyrin to form heme, a cofactor in hemoglobin and other hemoproteins. Thus, the major sources of iron used by bacterial pathogens *in vivo* are hemoglobin and non-heme iron-protein complexes [1]. Heme is a preferred iron source for some bacteria associated with mammals, including medically relevant Gram-positive pathogens *S. aureus* and *Streptococcus pyogenes* [2], [3].

The heme acquisition machineries in a number of Gram-positive bacteria have been described at least partially. The *S. aureus* heme uptake system is made of the surface proteins, IsdA, IsdB, and IsdC, IsdH, and the membrane transporter IsdDEF, where IsdE is the lipoprotein component [4], [5]. The heme acquisition machinery of *S. pyogenes* consists of the surface proteins, Shr and Shp, and the membrane transporter HtsABC [6]–[10]. *Streptococcus equi* and *Corynebacterium diphtheriae* use a system that is similar to the *S. pyogenes* system [11], [12], whereas *Bacillus anthracis* produces secreted proteins for heme uptake in addition to a homologue of the *S. aureus* IsdC protein [13]. Surface proteins involved in heme acquisition commonly contain one or more the NEAT (NEAr Transporter) domain(s) [14] that are involved in heme binding and/or protein interactions. Rapid heme transfer occurs specifically from one protein to another among methemoglobin (metHb) and the heme acquisition proteins of *S. aureus* and *S. pyogenes* [15]–[19], supporting heme acquisition pathway schemes of metHb → IsdB → IsdA → IsdC → IsdDEF in *S. aureus* [17] and metHb → Shr → Shp → HtsABC in *S. pyogenes* [19].

Significant progress has been made to understand the mechanism of rapid heme transfer from one protein to another. Ferric Shp transfers heme to apo-HtsA at a rate constant that is ∼4,000 times greater than the rate of simple heme dissociation from ferric Shp into solvent, and detailed kinetic analyses of Shp-apoHtsA heme transfer found that the heme transfer reactions follow a concerted two-step kinetic mechanism in which Shp first forms a complex with apoHtsA and then transfers its heme to apo-HtsA with a single kinetic phase [20]. The heme transfer from IsdA to IsdC follows a similar kinetic mechanism to that of the Shp/HtsA reaction [16]. Kinetic and spectroscopic analyses of the Shp/HtsA reaction using heme axial residue-to-alanine replacement protein variants of Shp and HtsA reveal a direct axial ligand replacement mechanism in which the HtsA axial residues M79 and H229 specifically displace the M66 and M153 residues of Shp, respectively [21]–[23]. A structural study on the metHb/IsdH complex supports a direct extraction of metHb heme by the heme-binding domain of IsdH [24].

IsdB acquires heme from metHb at a rate that is about ≥80 times greater than the rate of passive heme dissociation from metHb [17]. IsdB is comprised of two NEAT domains (N1 and N2) that divide the protein into 5 structural domains and/or segments. The N2 domain contains the heme-binding pocket of IsdB [18], and N1 is believed to capture metHb. The N2 domain alone cannot acquire heme from metHb [18]. Functional roles of the IsdB domains or segments other than N2 are not well understood. The purpose of this study is to elucidate the role of the domains and/or segments of IsdB in the acquisition of metHb heme. We have found that: 1) the domain between N1 and N2 enhances the affinity of N2 for heme by slowing down heme dissociation from N2 and favorably driving the metHb/IsdB heme transfer reaction; 2) IsdB N-terminal segment is required for the rapid and single phase kinetics of the metHb/IsdB heme transfer reaction; and 3) the C-terminal segment does not contribute to the kinetics and equilibrium of the metHb/IsdB reaction. Our findings support a heme transfer mechanism in which multiple IsdB domains and segments coordinate direct assimilation of heme from metHb.

## Results

### Recombinant IsdB Protein Constructs

**Figure 1:**
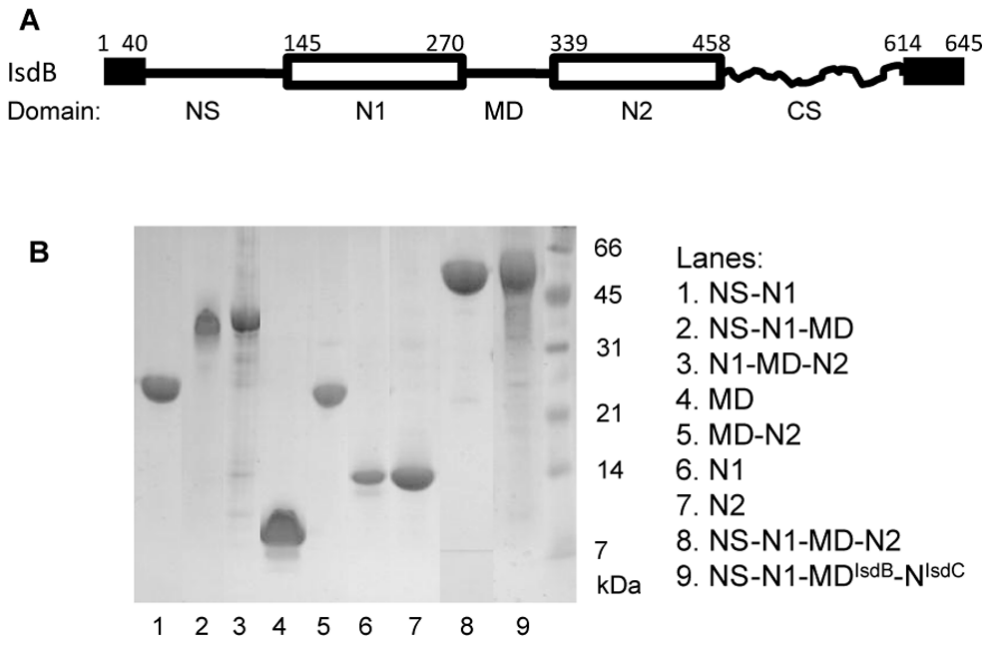
Recombinant IsdB fragments. (A) A schematic diagram depicting the domain and segment structures of IsdB. The protein contains the secretion signal sequence, N-terminal segment (NS), NEAT 1 (N1), middle (MD), and NEAT 2 (N2) domains, and C-terminal segment (CS), and cleaved transmembrane domain and charged tail. The numbers are the positions of amino acid residues at the start and end of the indicated domains and segments. (B) SDS-PAGE analysis of purified recombinant IsdB fragments or fusion protein.

The primary sequence of IsdB is comprised of an N-terminal secretion signal sequence, the mature IsdB protein, and a C-terminal region consisting of a transmembrane domain and a short C-terminal stretch rich in positively and negatively charged residues. The N- and C-terminal ends are both cleaved to generate the mature form of IsdB that is anchored to the cell wall at the newly processed C-terminal ends [25]. The mature IsdB protein is comprised of two NEAT domains (N1 and N2) that segment the protein into five distinct regions or domains: an N-terminal segment (NS) (amino acids 40–144); N1 (amino acids 145–270); a middle domain (MD) (amino acids 271–338); N2 (amino acids 339–458); and a C-terminal segment (CS) (amino acids 459–613) (Figure 1A) [14]. To identify IsdB domains and segments that critically contribute to the kinetics and equilibrium of the apo-IsdB/metHb reaction, we prepared the following recombinant IsdB fragments: N1, MD, N2, NS-N1, N1-MD, MD-N2, NS-N1-MD, N1-MD-N2, N1*-MD-N2 (amino acids 122–458), and NS-N1-MD-N2. We also fused NS-N1-MD of IsdB to the NEAT domain of IsdC (NS-N1-MD-N^IsdC^). All these fragments except N1 were expressed in soluble form and were tag-free. The MD-N2, N1-MD-N2, and NS-N1-MD-N2 fragments were produced both as heme-bound holo- and heme-free apo-forms, and the apo-form of each fragment could be separated from its holo-form by chromatography. These fragments were purified to >80% of purity according to SDS-PAGE analysis (Figure 1B). MD displayed a circular dichroism spectrum similar to the one reported by Spirig *et al*. [26] (data not shown). NMR analyses of ^15^N labeled N2, MD-N2, N1* (amino acids 122–270) and N1*-MD (containing amino acid residues 122 to 338) indicate that the protein constructs are well folded in solution, and the NMR-determined structures of N1* and N2 are overall similar to those of N2^IsdH^ and N2^IsdB^, respectively [27], [28], [29]. Functional characterizations of other IsdB protein fragments, described in this manuscript, support the notion that they adopt folds similar to the ones they possess in the full-length IsdB protein.

### N2 cannot rapidly and efficiently acquire heme from metHb but directly and efficiently transfers its heme to IsdC

**Figure 2:**
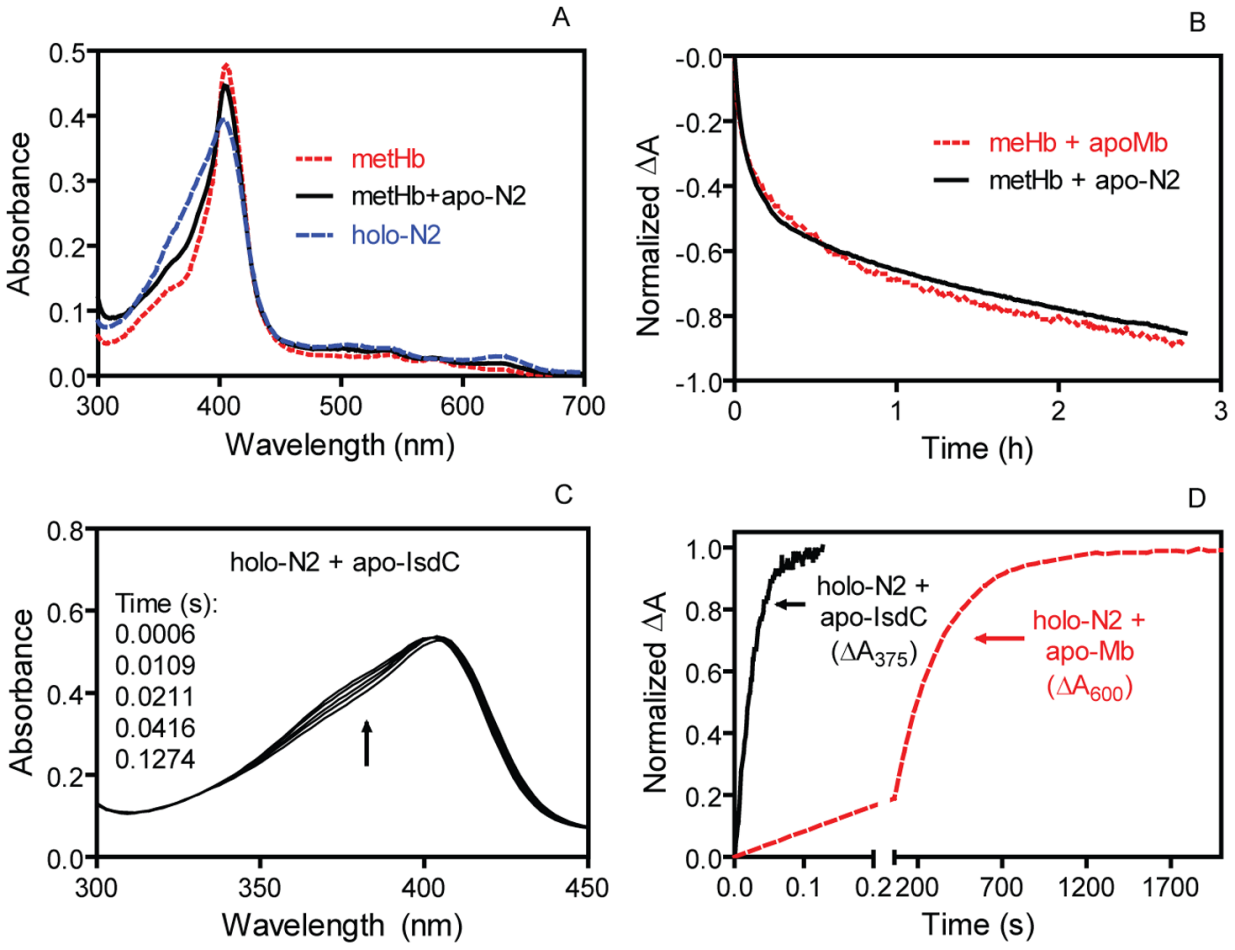
Slow and less favorable heme acquisition from metHb and rapid heme donation to IsdC by N2. (A) Absorption spectrum comparison of metHb (3 µM heme), metHb (3 µM heme)/30 µM apo-N2, and 3 µM holo-N2 after 12 h incubation at room temperature. (B) Time course of normalized ΔA at 406 nm associated with partial heme transfer from metHb (3 µM heme) to 30 µM apo-N2. Time course of normalized ΔA at 600 nm for the passive heme loss of metHb to apo-H64V/H68F myoglobin (40 µM) is included for comparison. (C) Absorption spectra of a mixture of 4 µM holo-N2 and 30 µM apo-IsdC as a function of time after mixing in a stopped-flow spectrophotometer. The arrow indicates the direction of the spectral shift with time. (D) Comparison of time course of normalized ΔA_375_ in the reaction of 4 µM holo-N2 with 30 µM apo-IsdC and ΔA_600_ in the reaction of 4 µM holo-N2 with 40 µM apo-H64V/H68F myoglobin.

To determine whether N2 retains the ability of IsdB to efficiently and rapidly acquire heme from metHb, the equilibrium and kinetics of the metHb/apo-N2 reaction were examined spectrally. Characteristic changes in absorption spectrum during the metHb/IsdB heme transfer reaction have been previously established [17]. Holo-N2 and holo-IsdB exhibit identical absorption spectrum (data not shown). A shift in the absorption spectrum of a metHb/apo-N2 reaction from the spectrum of metHb towards that of holo-N2 was used to assess heme transfer from metHb to apo-N2. Following 12 h incubation of 30 µM apo-N2 and 3 µM metHb, 10% of the metHb heme was transferred to N2 (Figure 2A) (Table 1), whereas 70% of metHb heme was transferred to full length IsdB under similar conditions (Table 1), indicating that the N2 reaction with metHb is less favored than the IsdB reaction with metHb. The two heme transfer reactions also display different kinetics. The metHb/apo-N2 reaction took several hours to complete and displayed biphasic kinetics with apparent rate constants of 0.0030 s^−1^ and 0.00019 s^−1^, which are close to the 0.0034 s^−1^ and 0.00020 s^−1^ rate constants of simple dissociation of heme from the β and α subunits of metHb measured using H64V/H68F apomyoglobin as a heme scavenger [30] (Figure 2B). In contrast, the metHb/apo-IsdB reaction was completed within 30 seconds following mixing and exhibits a single kinetic phase with an apparent rate constant of 0.3 s^−1^ under the same conditions (Table 1). These results indicate that N2 by itself can passively acquire a small portion of metHb heme.

**Table 1:** Apparent Rate Constants and Percentage of Heme Transferred in Various Reactions.

heme donor	heme acceptor	*k* or *k*_1_ (s^−1^)^a^	*k*_2_ (s^−1^)	*transferred heme%*
metHb	IsdB	0.30		71
	NS-N1-MD-N2	0.28		70
	N1-MD-N2	0.025	0.00191	64
	N1*-MD-N2	0.018	0.00265	66
	MD-N2	0.0031	0.00023	67
	N2	0.0030	0.00019	10
	N2/NS-N1-MD	0.21		78
	N2/NS-N1	0.0039	0.00047	15
	N2/NS-N1/MD	0.0034	0.00033	69
	N2/N1	0.0031		13
	N2/MD	0.0025	0.00024	68
	MD-N2/NS-N1	0.0046	0.00033	65
	NS-N1-MD-N^IsdC^	0.0041	0.00056	55
	N2 + NS-N1-MD-N^IsdC^	0.13		63
	H64Y/V68F Mb^b^	0.0034	0.00020	
	H64Y/V68F Mb/NS-N1-MD	0.0037	0.00040	
N2	H64Y/V68F Mb^b^	0.0034		
	H64Y/V68F Mb/NS-N1-MD^b^^,^ c	0.00014		

a The data were obtained using 3 µM heme donor and 30 µM each component in heme acceptor mixtures unless specified otherwise. Percentages of heme transferred were obtained at 30 min of the fast metHb/IsdB, metHb/NS-N1-MD-N2, and metHb/N2/NS-N1-MD reactions and at 12 h of the other slower reactions.

b 40 µM H64Y/V68F Mb was used.

c 8 µM NS-N1-MD was used.

To establish whether N2 alone can still directly transfer its heme to apo-IsdC, spectral change of a holo-N2/apo-IsdC reaction was monitored using a stopped-flow spectrophotometer. Heme transfer from holo-N2 to apo-IsdC was completed within 1 second following mixing (Figure. 2C), with an apparent rate constant of 39 s^−1^, which was 3.6 times greater than that of the heme transfer reaction from holo-IsdB to apo-IsdC (*k* ∼10.7 s^−1^) [17], and >10,000 fold greater than the 0.0037 s^−1^ rate constant for the simple dissociation of heme from holo-N2 as measured using apo-V64Y/V68F myoglobin as a heme scavenger (Figure 2D). These results indicate that holo-N2 retains the ability of IsdB to rapidly and directly transfer heme to IsdC.

### Minimal region of IsdB for rapid and single phase kinetics of the metHb/IsdB reaction

**Figure 3:**
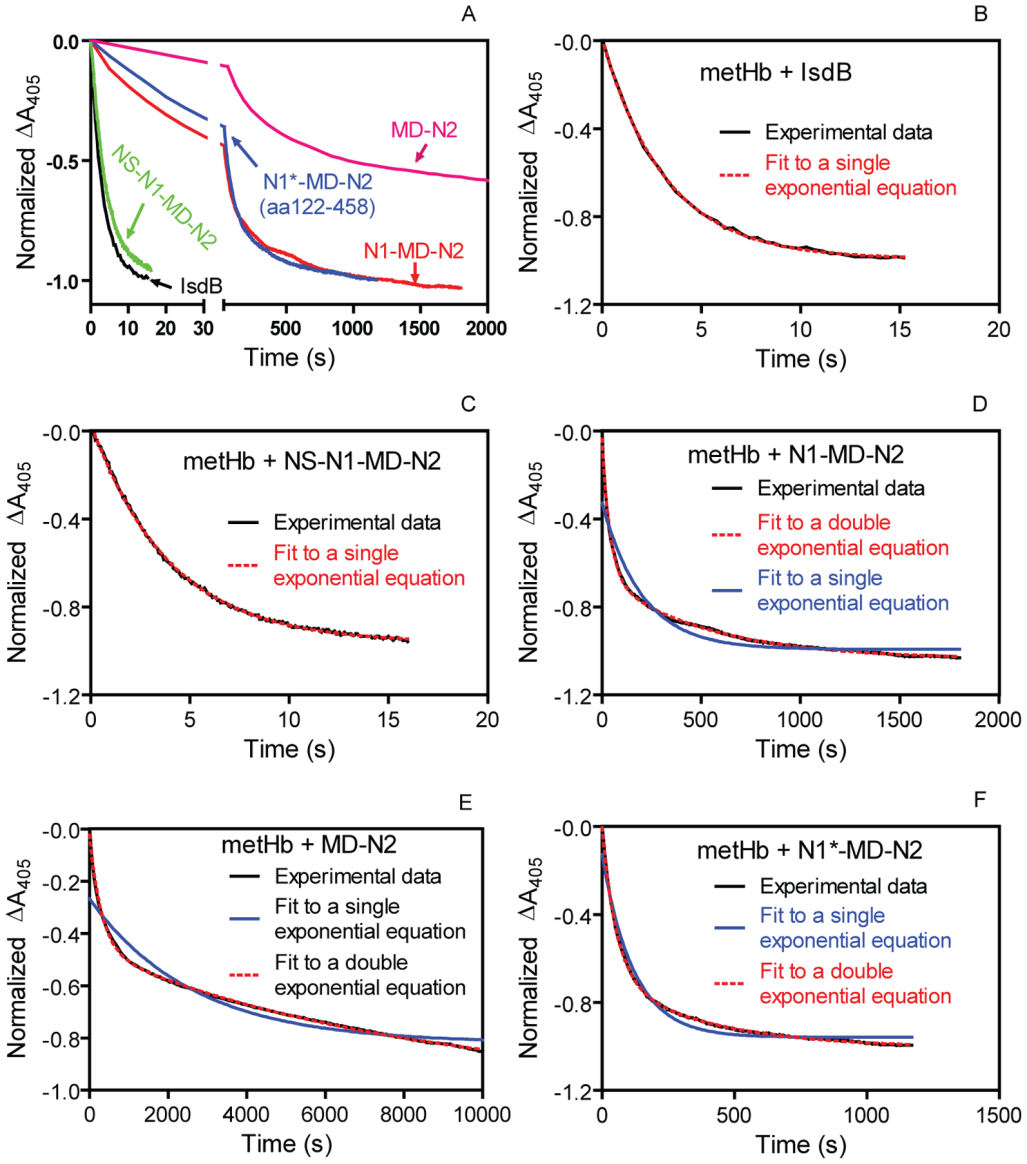
(A) Time courses of normalized ΔA_405_ in the reactions of metHb (containing 3 µM heme) with 30 µM IsdB, NS-N1-MD-N2, N1-MD-N2, N1*-MD-N2, and MD-N2. (B-F) Overlay of the experimental data (black curve) with curve(s) fit to a single or double exponential equation (red or blue curve).

Since the metHb/apo-N2 reaction was slower and less favorable than the metHb/IsdB reaction, we hypothesize that the rapid heme transfer from metHb to IsdB requires the assistance from IsdB domains and segments other than N2. We first tested this hypothesis by determining the minimal region of IsdB necessary for rapid and efficient heme acquisition from metHb. MetHb reacted with the apo-form of each of MD-N2, N1-MD-N2, NS-N1-MD-N2, and full-length IsdB. The percentages of metHb heme transferred to N1-MD-N2 and MD-N2 were 64% and 70%, respectively, which were comparable to 70% of metHb heme transferred in the metHb/IsdB reaction and were substantially higher than 10% of metHb heme transferred in the metHb/N2 reaction (Table 1). These reactions displayed different kinetic patterns with different observed rate constant(s). The time course of ΔA_405_ in each reaction was measured and fit to an exponential expression to obtain observed constant(s) for heme transfer reactions. The metHb/NS-N1-MD-N2 and metHb/IsdB reactions had very similar time course of ΔA_405_ (Figure 3A) and both displayed a single kinetic phase with similar observed rate constants (Figure 3B and 3C) (Table 1). Sequential deletions of NS and N1 progressively reduced the rates of the heme acquisition from metHb (Figure 3A) and the deletions both changed the kinetic pattern from the single kinetic phase to double kinetic phases in which the fitting curves from a double exponential equation but not from a single exponential equation overlay perfectly with the experimental data (Figure 3D and 3E). The observed rate constant for the fast phase of the metHb/N1-MD-N2 was one eleventh of that in the metHb/NS-N1-MD-N2 reaction but 8 times greater than that of the fast phase of the metHb/MD-N2 reaction (Table 1). The predicted N1 domain contains amino acid residues 145–270 [14], and the Clubb group used 20 extra amino acid residues for the N1 domain in a recent study [26]. To determine whether these extra residues contribute to the kinetics of the metHb/IsdB reaction, we prepared an N1*-MD-N2 fragment containing amino acid residues 122 to 458. The metHb/N1*-MD-N2 and metHb/N1-MD-N2 reactions displayed similar time courses of ΔA_405_ and both exhibited a double kinetic phases with similar observed rate constants (Figures 3A, 3D and 3F) (Table 1). These results indicate that NS-N1-MD-N2 is the minimal region necessary for the rapid heme transfer and the single phase kinetics and that the region containing the amino acids 40-121 of the NS segment is critical for the rapidness and single phase kinetics of the metHb/IsdB reaction. These data also indicate that the CS region of IsdB is not directly involved in the heme transfer reaction from metHb to IsdB.

### *In trans* rescue of rapid and efficient heme transfer from metHb to N2 by ND-N1-MD

**Figure 4:**
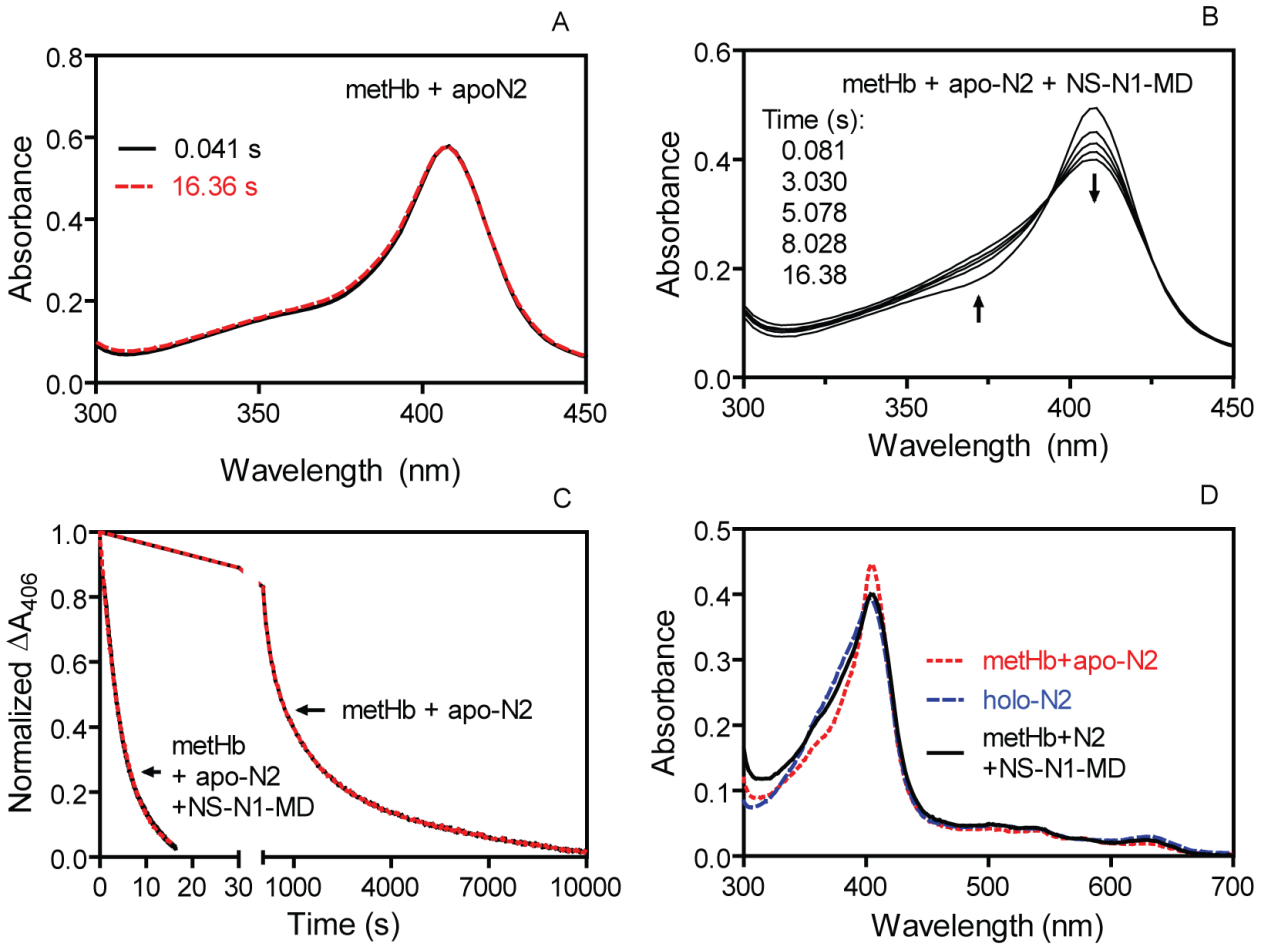
NS-N1-MD enhances the rate and extent of heme transfer from metHb to N2. (A) No shift in the spectrum of a mixture of 3.0 µM metHb heme and 30 µM apo-N2 at the indicated times after mixing in a stopped-flow spectrophotometer. (B) Shift of absorption spectra after mixing 3 µM metHb heme with 30 µM apo-N2 and 30 µM NS-N1-MD. The arrows indicate the directions of the spectral shift during the reaction. (C) Time course of normalized spectral changes in the 3.0 µM metHb heme/30 µM apo-N2 reactions in the absence and presence of 30 µM NS-N1-MD. Black lines are the experimental data, and the dotted red lines are curves of fitting the metHb/N2/NS-N1-MD and metHb/N2 reactions data to single and double exponential equations, respectively. (D) Overlay of the absorption spectra of metHb/apo-N2 after 12-h incubation, holo-N2, and metHb/apo-N2/NS-N1-MD after 30-min incubation.

Since NS-N1-MD is required for the rapid and favorable heme acquisition from metHb by NS-N1-MD-N2, we tested whether NS-N1-MD *in trans* enhances the rate of the heme transfer and percentage of heme transferred from metHb to N2. As measured using a stopped-flow spectrophotometer, no heme transfer from metHb to apo-N2 was detectable within the timeframe of the apo-IsdB/metHb reaction (Figure 4A). However, following the mixing of 3 µM metHb with a 30 µM NS-N1-MD/30 µM N2 solution, the spectrum of the mixture shifted from that of metHb toward that of holo-N2 within 30 seconds (Figure 4B). Calculation based on the spectral data indicated that 78% of metHb heme was transferred to N2 in the presence of NS-N1-MD. The NS-N1-MD fragment itself did not change the spectrum of metHb (Data not shown). Spectral changes in the NS-N1-MD-rescued heme transfer reaction monitored over time fit to a single exponential equation, yielding an observed rate constant of 0.21 s^−1^, which was close to the apparent rate constant of 0.30 s^−1^ of the metHb/apo-IsdB reaction but was >65-fold greater than the observed rate constants of heme transfer from metHb to apo-N2 alone (*k* = 0.0034 s^−1^ and 0.00020 s^−1^) under the same conditions (Figure 4C). Thus, NS-N1-MD transforms the indirect, slow, and kinetically biphasic metHb-to-N2 heme transfer reaction into a rapid reaction with a single kinetic phase, and this fragment also shifts the equilibrium of the reaction in favor of the formation of holo-N2 as shown in Figure 4D in which the spectrum of the metHb/apo-N2/NS-N1-MD reaction shifted from that of the metHb/apo-N2 reaction towards that of holo-N2.

### MD alters the equilibrium of the metHb/apo-N2 reaction

**Figure 5:**
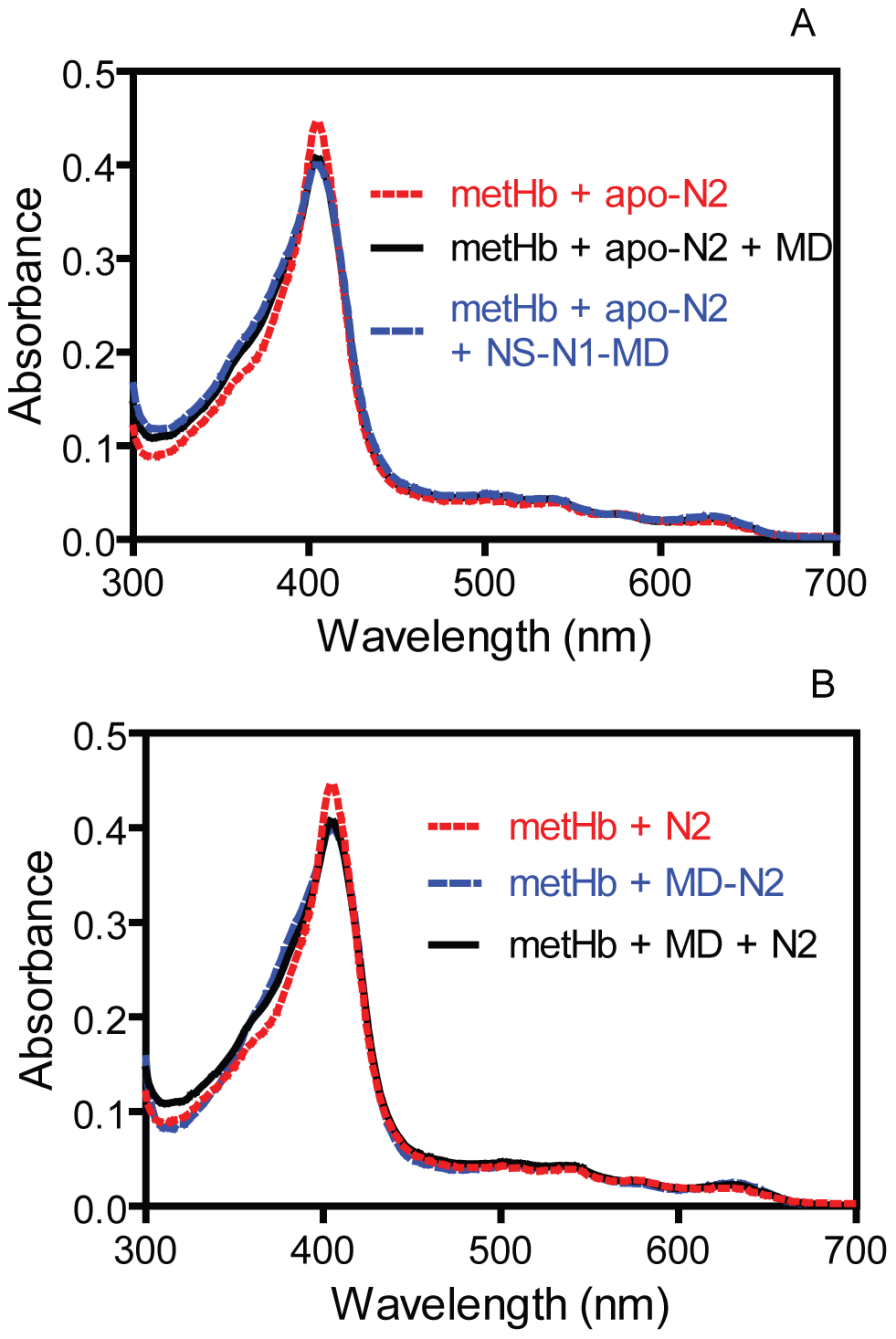
Effect of MD on the equilibrium of the metHb/apo-N2 reaction. (A) Overlay of the spectra of the metHb/apo-N2, metHb/apo-N2/MD, and metHb/apo-N2/NS-N1-MD. (B) Overlay of the spectra of the metHb/apo-N2, metHb/apo-MD-N2, and metHb/apo-N2/MD. MetHb in all the reactions contained 3 µM heme, and the concentration of each of the other components was 30 µM.

To identify the domain or segment in the N-terminal region that shifts the equilibrium of the metHb/apo-N2 reaction, spectra of the metHb/apo-N2 reactions in the presence of NS-N1-MD or MD were recorded and percentages of metHb heme transferred were calculated. The metHb/apo-N2/NS-N1-MD and metHb/apo-N2/MD reactions exhibited similar final spectral profiles (Figure 5A) corresponding to 78% and 68% of heme transferred from metHb to N2, respectively. A metHb/apo-N2/NS-N1 reaction transferred 15% of heme from metHb to N2 that was similar to 10% of metHb heme transferred in the metHb/apo-N2 reaction (Table 1). These data indicate that MD, but not NS-N1, is responsible for the efficient 65% heme transfer from metHb to IsdB. To determine whether covalent linkage between MD and N2 is important to favorably drive the metHb/IsdB reaction, the metHb/apo-N2/MD and metHb/apo-MD-N2 reactions were compared. The two reactions displayed similar final spectral profiles with nearly identical percentages of metHb heme transferred (Figure 5B) (Table 1). Therefore, the covalent linkage between MD and N2 does not appear to be critical for the function of MD to favorably drive the heme transfer reaction from metHb to N2, suggesting that the MD function is mainly mediated by protein-protein interactions between MD and N2.

### NS-N1-MD and MD *in trans* reduce the rate of passive heme dissociation from holo-N2 but not from metHb

**Figure 6:**
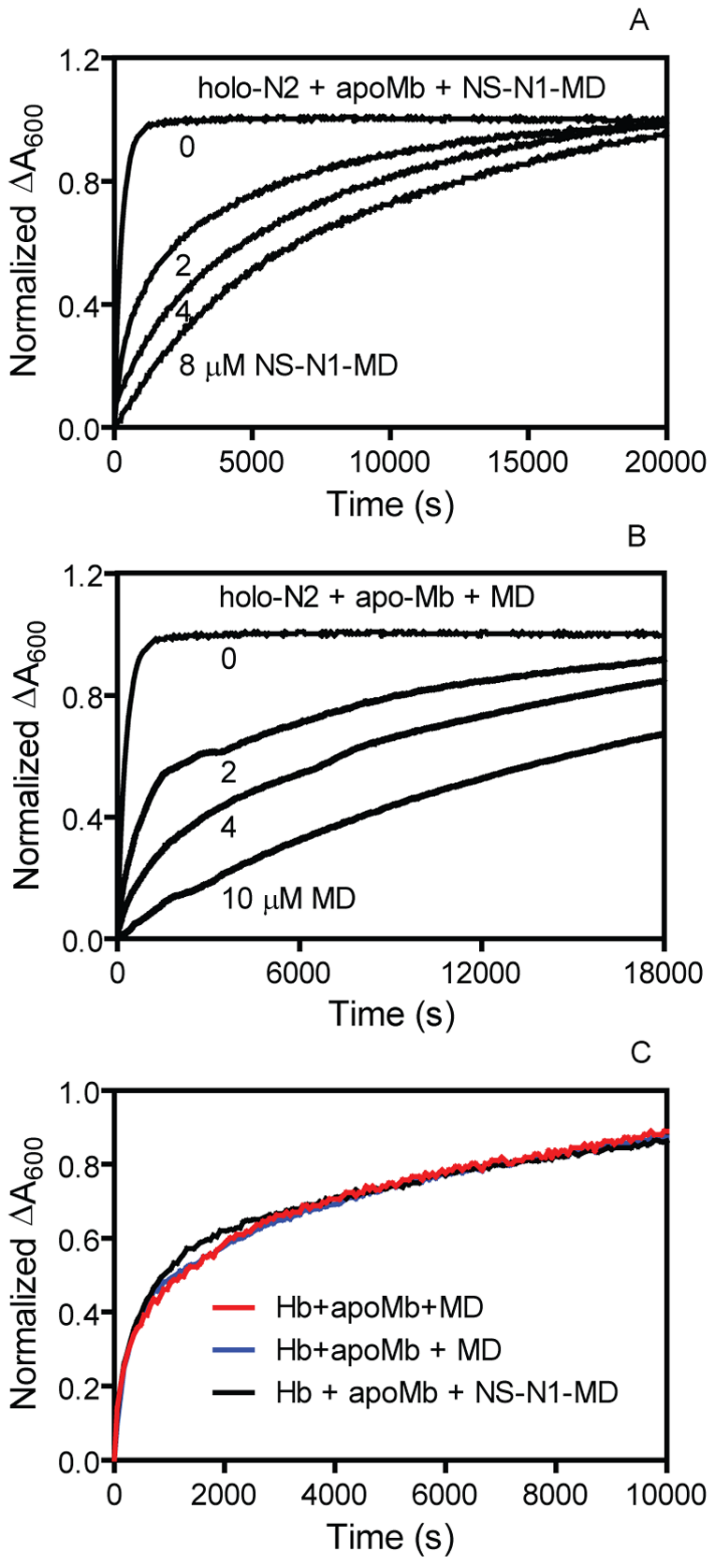
Effects of NS-N1-MD and MD on heme dissociation from holo-N2. (A and B) Time courses of normalized ΔA_600_ measuring heme dissociation from holo-N2 using 40 µM apo-H64V/H68F myoglobin as a heme scavenger in the presence of various NS-N1-MD (A) or MD (B) concentrations. (C) Heme dissociation from metHb in the absence and presence of 30 µM NS-N1-MD or MD as measured using apo-H64V/H68F myoglobin as heme scavenger.

We hypothesized that NS-N1-MD and MD must be able to alter the affinity of N2 or of metHb for heme in order to influence the equilibrium of the heme transfer reaction. To test this hypothesis, we examined the effect of NS-N1-MD and MD on the passive heme dissociation from holo-N2 and metHb using apo-H64Y/V68F myoglobin as a heme scavenger. Heme dissociation from holo-N2 slowed down in a dose-dependent manner as a function of increased NS-N1-MD or MD concentrations (Figure 6A and 6B). In contrast, NS-N1-MD and MD had no effect on the rate of passive heme dissociation from metHb (Figure 6C). Spectral changes monitoring the time course of heme dissociation from holo-N2 in the presence of 0 µM and 8 µM NS-N1-MD were fit to a single exponential equation, but could not be fit to a single exponential equation in the presence of 2 µM and 4 µM NS-N1-MD, indicating that holo-N2 was nearly all in complex with NS-N1-MD at 8 µM, but not at 2 µM and 4 µM MD. This indicates that the heme dissociation constant for a MD/holo-N2 complex is in the µM range. Fitting of the spectral changes leads to observed rate constants for heme dissociation at 0 µM and 8 µM ND-N1-MD of 3.5×10^−3^ s^−1^ and 1.3×10^−4^ s^−1^, respectively, indicating that the presence of NS-N1-MD reduces the observed rate constant of heme dissociation from N2 by 96%. These data suggest that the interaction of MD with N2 increases the affinity of N2 for heme by slowing down the rate of heme dissociation from N2 and that MD has no effect on heme dissociation from metHb.

### MD-N2 had higher affinity for heme than N2

**Figure 7:**
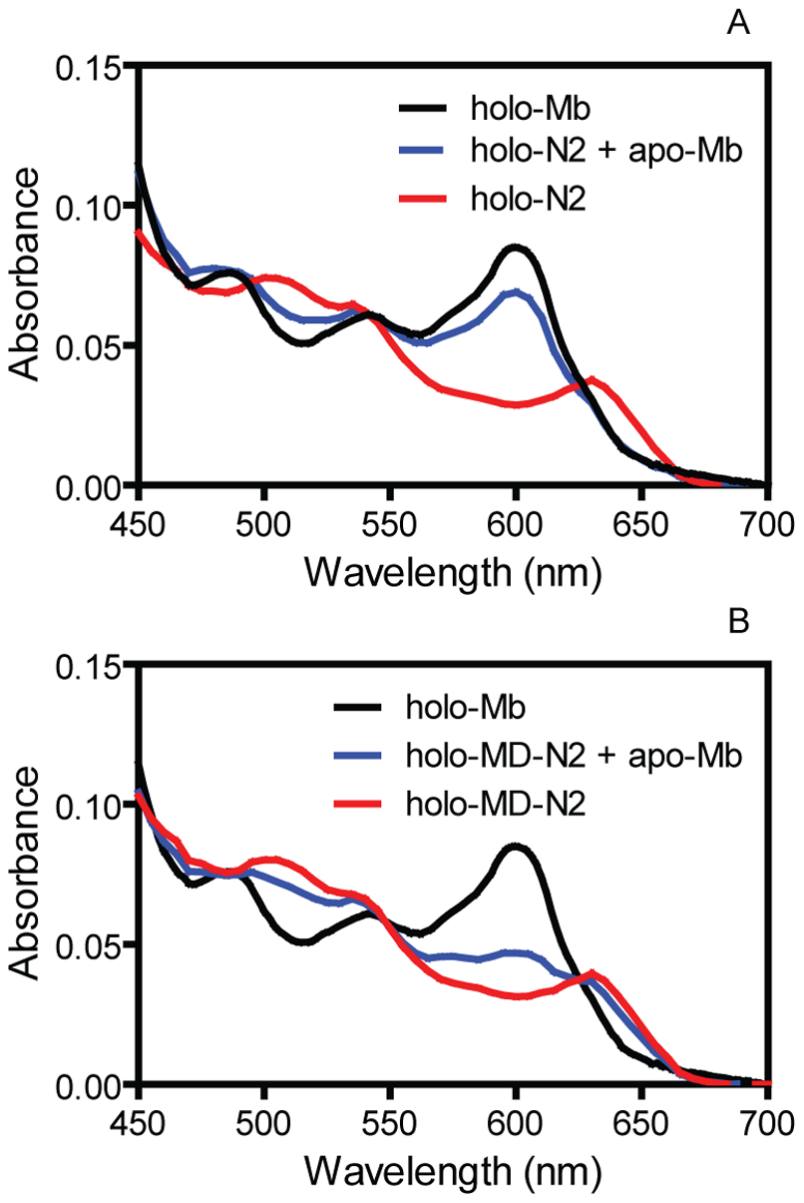
Qualitative evidence for higher affinity of MD-N2 for heme than N2. (A) Overlay of the spectra of 8 µM holo-N2, 8 µM holo-H64V/H68F myoglobin, and a mixture of 8 µM holo-N2 and 40 µM apo-H64V/H68F myoglobin. (B) Overlay of the spectra of 8 µM holo-MD-N2, 8 µM holo-H64V/H68F myoglobin, and a mixture of 8 µM holo-MD-N2 and 40 µM apo-H64V/H68F myoglobin. All the spectra were taken after 12-h incubation of the solutions at room temperature.

The effects of MD on the equilibrium of the metHb/apo-N2 reaction and the rate of heme dissociation from holo-N2 suggest that MD enhances the affinity of N2 for heme. We first used a UV-Vis spectroscopic approach to compare the affinity of N2 and MD-N2; however, this approach was not sufficiently sensitive to measure the affinity of N2 and MD-N2 for heme because both had high affinity for heme. We then tried to measure the affinity of N2 and MD-N2 by determining the kinetics of heme binding to and dissociation from them but the approach did not work either because the loss of heme from holo-N2 and, especially, holo-MD-N2 to myoglobin was not complete. Thus, we used the reactions of apo-H64Y/V68F myoglobin with holo-N2 and holo-MD-N2 to qualitatively compare the relative affinity of N2 and MD-N2 for heme. The majority of N2-associated heme was lost to apo-H64Y/V68F myoglobin (Figure 7A) whereas just a small portion of MD-N2-associated heme was lost to the apo-myoglobin protein (Figure 7B). From these data, we conclude that MD-N2 has a higher binding affinity for heme than N2, supporting the function of MD as enhancing the affinity of IsdB for heme.

### NS-N1 and its linkage to MD are needed to enhance the rate of heme transfer from metHb to apo-N2

The critical role of NS and N1 in the kinetics of the heme transfer reaction is supported by the observation that rapid heme transfer can be reconstituted from metHb, N2, and NS-N1-MD. We next determined whether a mixture of the NS-N1 and MD fragments are able to enhance the rates of heme transfer from metHb to apo-N2. Similar to the metHb/apo-N2 reaction, the metHb/apo-N2/NS-N1/MD reaction displayed biphasic time course of ΔA_405_, with two apparent rate constants of 0.0034 s^−1^ and 0.00033 s^−1^ that were very similar to the observed rate constants of 0.0030 s^−1^ and 0.00019 s^−1^ of the metHb/apo-N2 reaction (Table 1). Similarly, NS-N1 alone could not enhance the rates of heme transfer in the metHb/apo-MD-N2/NS-N1 reaction (Table 1), indicating that covalent linkage between NS-N1 and MD is crucial for NS-N1 to facilitate the rapid heme transfer between metHb and N2.

### NS-N1-MD-N^IsdC^ slowly acquires heme from metHb but enhances the loss of heme from metHb in the presence of N2

**Figure 8:**
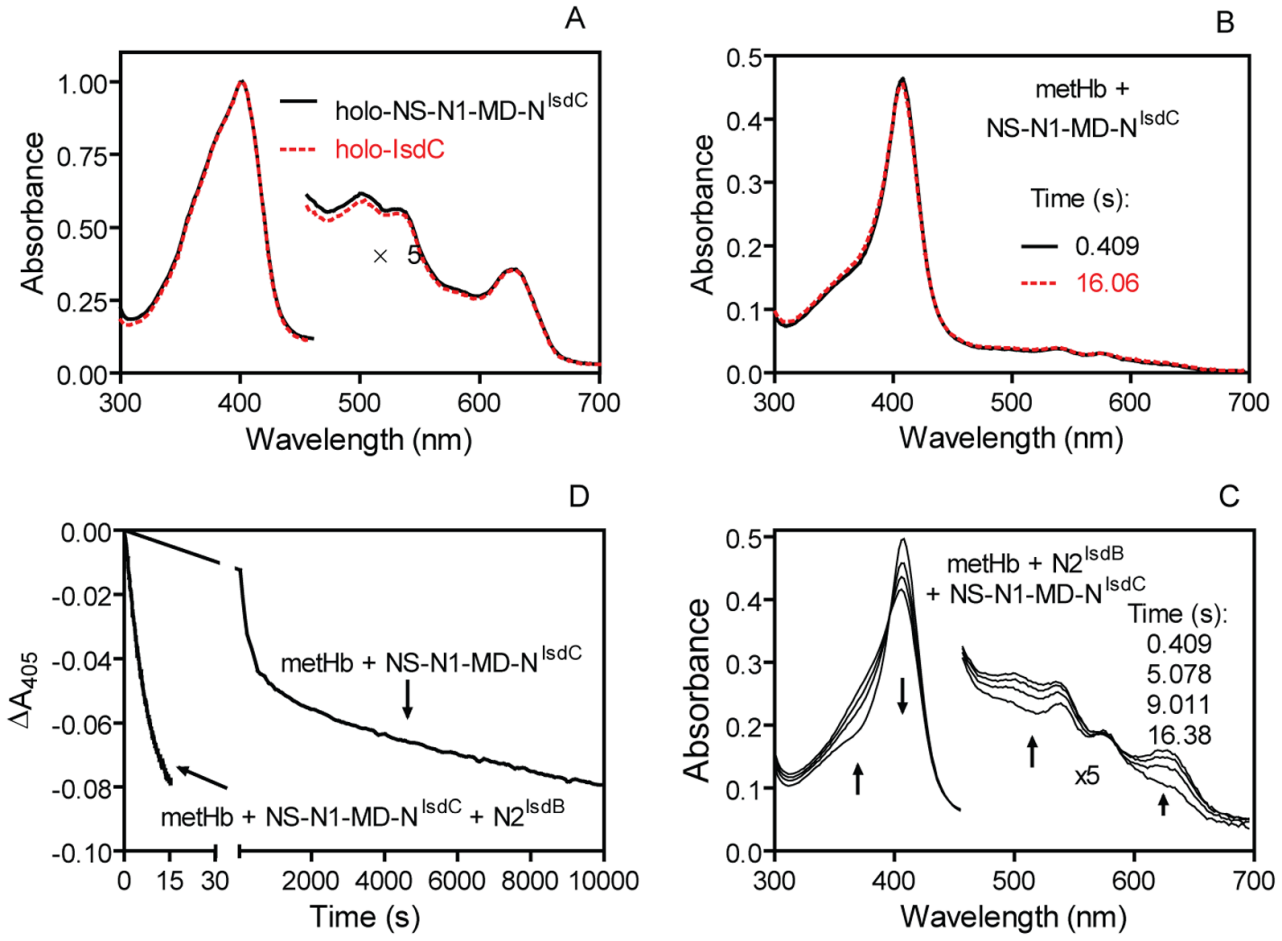
Inability of the NS-N1-MD-N^IsdC^ fusion protein to rapidly acquire heme from metHb. (A) Overlay of the absorption spectra of holo-NS-N1-MD-N^IsdC^ and holo-IsdC. (B) No shift in the spectrum of a mixture of 3.0 µM metHb heme and 30 µM apo-NS-N1-MD-N^IsdC^ at the indicated times after mixing in a stopped-flow spectrophotometer. (C) Shift of absorption spectra after mixing 3 µM metHb heme with 30 µM apo-N2 and 30 µM NS-N1-MD-N^IsdC^. The arrows indicate the directions of the spectral shift during the reaction. (D) Time course of ΔA_405_ in the 3.0 µM metHb heme/30 µM apo-N2 reactions in the absence and presence of 30 µM NS-N1-MD-N^IsdC^.

To determine whether the truncated NS-N1-MD fragment of IsdB can enhance heme transfer from metHb to NEAT domains of other Isd proteins, we investigated the heme transfer from metHb to NS-N1-MD-N^IsdC^. The bound heme in the fusion protein displayed an absorption spectrum nearly identical to that of holo-IsdC (Figure 8A), indicating that the structure of N^IsdC^ in the fusion protein is comparable to its fold in the full-length IsdC protein. As monitored by stopped-flow spectrophotometer, no obvious spectral changes were observed in the metHb/NS-N1-MD-N^IsdC^ reaction in the first 16 s (Figure 8B), indicating that the fusion protein cannot rapidly acquire heme from metHb. However, we observed that the fusion protein could eventually capture heme from metHb as the reaction progressed (Figure 8C), with observed rate constants of 0.0041 s^−1^ and 0.00056 s^−1^ of the heme transfer from metHb to the fusion protein (Table 1), which were similar to heme dissociation rate constants measured for heme transfer from metHb to heme scavenger apo-H64Y/V68F myoglobin (Table 1). These results indicate that NS-N1-MD-N^IsdC^ can only capture heme that is spontaneously released from metHb. However, it is possible that the NS-N1-MD fragment in the fusion protein may have lost its ability to enhance the rate of heme transfer. To rule out this possibility, metHb was mixed with N2 and NS-N1-MD-N^IsdC^, and the spectrum of the reaction mixture rapidly shifted with time (Figure 8C), indicating that metHb rapidly lost its heme in this reaction. Spectral changes associated with the loss of heme from metHb in the metHb/NS-N1-MD-N^IsdC^/N2 reaction displayed single exponential kinetics with an apparent heme transfer rate constant of 0.13 s^−1^ (Figure 8D). Thus, the NS-N1-MD portion in the NS-N1-MD-N^IsdC^ fusion protein did not lose its function. Because holo-N2 rapidly transfers its heme to IsdC, heme transferred from metHb to N2 in the presence of NS-N1-MD-N^IsdC^ most likely ends up in the N^IsdC^ portion of the fusion protein. These results indicate that NS-N1-MD specifically enhances the rate of heme transfer from metHb to IsdB N2 but not to NEAT domains of other Isd proteins such as IsdC.

## Discussion

This report presents our findings on the role of individual domains and segments of IsdB in the kinetics and equilibrium of heme transfer from metHb to IsdB. First, we find that the MD domain reduces the rate of heme dissociation from N2 and enhances the affinity of N2 for heme, favorably driving the equilibrium of the metHb/IsdB reaction. Secondly, both the NS and N1 regions critically contribute to the rapid kinetics of the heme transfer reaction, and NS is critical for the single phase kinetic pattern of the metHb/IsdB reaction. Thirdly, the rapid metHb heme acquisition by IsdB can be reconstituted from N2 and NS-N1-MD but not from N2, NS-N1, and MD. We also find that fusion of NS-N1-MD to N^IsdC^ does not enhance the rate of heme transfer from metHb to N^IsdC^. These findings identify protein regions of IsdB that are required for the rapid kinetics and favorable equilibrium of heme acquisition from metHb, providing new insights into the mechanism of heme assimilation from metHb by IsdB.

IsdB^N2N3^, a fragment of IsdB that contains amino acids 121–458 and lacks the CS region and part of NS, acquires heme from metHb at a rate faster than the rates of passive heme dissociation from metHb [26]. However, this rate is about one-sixth the rate reported for the metHb/IsdB heme transfer reaction [17], leaving questions as to whether the missing NS and CS regions of IsdB contribute to the heme acquisition reaction. In this study we found that the rate of heme transfer and percentage of metHb heme transferred in the metHb/NS-N1-MD-N2 reaction were comparable to those in the metHb/IsdB reaction. Thus, the CS region of IsdB is dispensable for rapid heme acquisition from metHb, indicating that CS is not directly involved in the heme acquisition reaction. IsdA and IsdC also possess a CS region C-terminal to their heme-binding NEAT domain. The CS regions of IsdB, IsdA, and IsdC are comprised of 155, 132, and 42 amino acids, respectively, and all these proteins are anchored to the bacterial cell wall at the C-terminal end of their CS region [25]. The different lengths of the CS region in these proteins would position these proteins sequentially through the cell wall envelope of *S. aureus*, suggesting that the CS region of the Isd proteins functions as a spacer to position these proteins or their heme-accepting domain appropriately across the cell wall envelope to enable the sequential relay of heme across the cell wall along the Isd heme acquisition pathway.

A novel finding of this study is the elucidation of the functional role of the MD domain in favorably driving the equilibrium of the metHb/IsdB reaction. The minimal region of IsdB necessary for the favorable equilibrium of the metHb/IsdB reaction is MD-N2. MD *in trans* enhances the percentage of metHb heme transferred to N2 and slows down the dissociation of heme from N2. These data indicate that the affinity of the heme-binding domain of IsdB for heme is not sufficiently strong for significant heme acquisition from metHb and that the MD domain drives the equilibrium of the reaction toward the formation of holo-IsdB by increasing the affinity of IsdB for heme. The MD domain of IsdB is homologous in sequence to the linker between N2 and N3 of IsdH, which is required for rapid acquisition of metHb heme by IsdH^N2N3^ [26]. Based on the structure of the metHb/IsdH^N2N3^ complex, IsdH linker and N2 position the N3 domain to facilitate IsdH-mediated heme capture [24]. IsdB MD most likely plays a similar role in the metHb/IsdB reaction. However, IsdB MD apparently has the additional role of increasing the affinity of IsdB for heme and favorably driving the equilibrium of the metHb/IsdB reaction. The finding that MD can function *in trans* implies that MD can interact with N2 of IsdB. The N2-linker fragment of IsdH does not rescue the slow heme transfer from metHb to N3 [26], which is consistent with the structure of the metHb/IsdH^N2N3^ complex in which the linker does not have significant interactions with N3 [24]. Our data suggest that the effect of MD on the affinity of IsdB for heme and equilibrium of heme transfer is unique to IsdB.

The role of MD in favorably driving the equilibrium of the metHb/IsdB reaction may be the basis for why IsdB can efficiently relay heme from metHb to IsdA. Heme donor and acceptor must have at least comparable affinity for heme in order for the acceptor to capture heme from the donor. MetHb can indirectly donate the majority of its heme to myoglobin but not to IsdA [17]. In contrast, IsdB efficiently acquires heme from metHb [17], suggesting that the affinity of IsdB for heme is higher than that of IsdA. Thus, we would expect that IsdA could not significantly acquire heme from IsdB. However, IsdA can assimilate most of IsdB heme [17]. This suggests that IsdB MD functions as an affinity switch altering the affinity of IsdB for heme. In such a switch mechanism IsdB MD interacts with N2 and increases the affinity of N2 for heme to allow efficient heme acquisition from metHb by IsdB, whereas in the IsdB/IsdA reaction, interactions of IsdA with IsdB disrupt the interaction between MD and N2 and reduce the affinity of IsdB for heme to allow heme transfer from IsdB to IsdA. The structural basis for this affinity switching function of IsdB MD is currently under investigation.

Another novel finding in this study is that the NS region of IsdB is required for the rapidness and single phase kinetic pattern of the metHb/IsdB reaction. IsdH^N2N3^ containing N2-linker-N3 of IsdH acquires heme from metHb at a rate that is similar to that of the reaction of metHb with IsdB^N1N2^ protein construct that contains amino acid residues 121–458 [26], which is one residue longer than our N1*-MD-N2. The apparent rate constants of the metHb/IsdB^N1N2^ or N1*-MD-N2 reaction in the study of Spirig *et al.* and our test are similar and are about one sixth of that of the metHb/IsdB reaction. More importantly, the metHb/N1*-MD-N2 reaction displays double phase kinetics in the time course of the spectral changes, but the metHb/full length IsdB and metHb/NS-N1-MD-N2 reactions display a single phase kinetics. The metHb/IsdH^N2N3^ reaction also displays double phase kinetics (unpublished data of B. Lei and R. Clubb). It is currently unknown whether the metHb/full length IsdH reaction is a faster reaction with single phase kinetics than the metHb/IsdH^N2N3^ reaction. It should be noted that the metHb/IsdB^N1N2^ and metHb/IsdH^N2N3^ reactions both display single phase kinetics in the study of Spirig *et al.* [26]. The discrepancy in the kinetic pattern between the studies of Spirig *et al.* and this study may be because the reactions were monitored for different time lengths in the two studies.

Kinetic analysis has been used to elucidate the mechanism of heme transfer from Shp to HtsA in which Shp first forms a complex with apo-HtsA and subsequently transfers its heme to HtsA [20]. The heme transfer from IsdA to IsdC follows a similar kinetic mechanism [16]. Subsequent kinetic and spectral studies using axial ligand mutants of Shp and HtsA have established a mechanism for the specific displacement of the Shp axial residues with the HtsA axial residues [21], [23]. The results in this study support the idea that the metHb/IsdB reaction follows the mechanism of direct metHb heme extraction by IsdB. MetHb can passively dissociate its heme, and the passive dissociation of metHb heme is a kinetically biphasic process due to the rates of heme dissociation from the α and β subunits of metHb being different [28]. The metHb/IsdB reaction is not only 88- and 2200-time faster than the rates of passive heme dissociation from the β and α subunits of metHb, respectively, but also possesses single phase kinetic characteristics [20]. The difference in the kinetic pattern between the metHb/myoglobin and metHb/IsdB reactions strongly suggests that the metHb/IsdB reaction does not follow a mechanism that involves the dissociation of heme from metHb followed by subsequent scavenging of the dissociated heme by IsdB. This interpretation is further supported by our results that IsdB NS-N1-MD region facilitates the acquisition of metHb heme by N2 but does not enhance heme dissociation from metHb. Furthermore, IsdB NS-N1-MD segment cannot enhance heme transfer from metHb to N^IsdC^ in the ND-N1-MD-N^IsdC^ fusion protein but can enhance the rate of heme loss from metHb in the presence of N2. These results imply that N2 is directly involved in heme acquisition from metHb. However, more investigations are needed to establish whether the metHb/IsdB reaction follows a mechanism of axial residue displacement similar to that of the Shp/HtsA reaction.

The linker between N2 and N3 of IsdH is α helical and, based on a structural modeling for the interactions between IsdH^N2-Linker^ and metHb, it has been proposed that IsdH N2-linker and IsdB N1-linker or N1-MD interact with and dissociate the quaternary structure of metHb to facilitate heme acquisition [26]. It turns out that IsdH N2-Linker actually positions N3^IsdH^ along the heme pocket in metHb to facilitate assimilation of metHb heme based on the structure of the metHb/IsdH^N2N3^ complex [24]. It is likely that IsdB N1-MD-N2 interacts with metHb in an analogous pattern due to its close sequence homology with IsdH N2-linker-N3 [26]. Indeed, residues F164 and Y167 of IsdB N1 appear to be critical for the binding of metHb to *S. aureus* [31]. Due to the current lack of structural information for IsdB NS, the structural basis for the role of NS in the heme transfer reaction between metHb and IsdB is unknown. We speculate that NS may cause dissociation of the quaternary structure of metHb. Alternatively, NS could enhance the interaction between N1 and metHb. Similarly, it is unknown whether the N-terminal region of IsdH including its N1 domain further enhances the rate and kinetics of heme transfer from metHb to IsdH.

Our data also suggest that intra-protein interaction among the IsdB domains and inter-protein interactions between metHb and IsdB domains and segments play critical roles in the kinetics and equilibrium of the metHb/IsdB heme transfer reaction. Critical intra-protein interaction within IsdB must be that occurring between IsdB MD and N2 domains. This interaction is sufficiently strong to alter the heme-binding affinity of N2 and to mediate the effect of NS-N1 on the kinetics of the metHb/N2 reaction. In our study, the NS-N1 fragment could not enhance the rate of heme transfer from metHb to MD-N2, indicating that strong interactions between NS-N1 and MD-N2 are lacking. Therefore, NS-N1 must interact directly with metHb to enhance the heme transfer rate. ND-N1-MD-N^IsdC^ could not rapidly acquire heme from metHb, suggesting that the rapid metHb/IsdB reaction also requires specific protein interaction between metHb and N2. These metHb/N2 interactions may be promoted by NS and N1 working together to facilitate metHb-to-N2 heme transfer.

**Figure 9:**
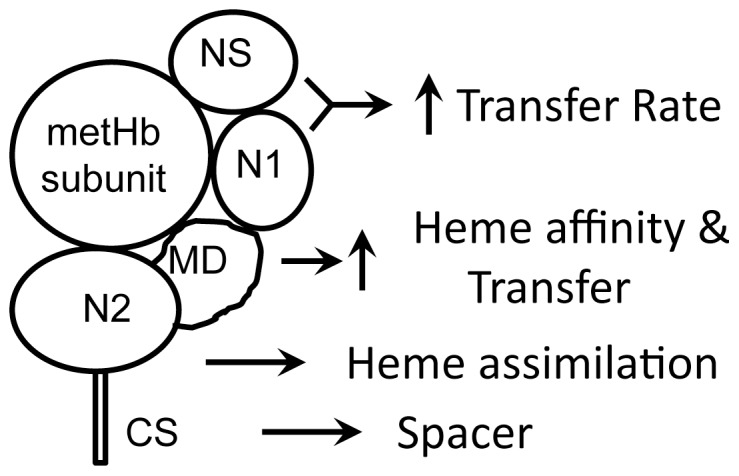
A schematic model depicting the distinct functions of individual domains of IsdB for the kinetics and equilibrium of the metHb/IsdB heme transfer reaction. In this model, NS-N1 specifically enhances direct heme assimilation from α and β subunits of metHb by N2; MD enhances the affinity of N2 for heme and favorably drives the equilibrium of the heme transfer reaction; and CS functions as a spacer to place IsdB at an appropriate position in the cell wall envelope of *S. aureus* for heme relay by the Isd heme acquisition system.

In summary, our findings support distinct functional roles for the different domains and segments of IsdB in the metHb/IsdB heme transfer reaction, and a model depicting how they participate in the direct acquisition of heme from metHb is proposed in Figure 9. This model shares some similarity with the one proposed for the metHb/IsdH reaction based on the structure of the metHb/IsdH^N2N3^ complex [24]. However, the model depicted in Figure 7 for IsdB contains the following unique information: The MD domain of IsdB interacts with and enhances the affinity of N2 to thermodynamically drive heme transfer from metHb to IsdB, and the NS-N1 region, but not just N1, enhances specific metHb/N2 interactions to facilitate direct heme extraction by N2 from the heme pocket in metHb.

## Materials and Methods

### Gene Cloning

The two NEAT domains (N1 and N2) of IsdB divide the protein into 5 separate regions or domains, namely the N-terminal segment (NS), NEAT 1 (N1), middle (MD), and NEAT 2 (N2) domains, and C-terminal segment (CS) (Fig. 1A). DNA fragments of the *isdB* gene encoding truncated constructs of IsdB containing various domains and sements were amplified by PCR using the cloned *isdB* gene [17] as template and the primers listed in Table 2. The primers for fragments are: N1, 1 and 5; MD, 3 and 6; N2, 4 and 7; NS-N1, 1 and 5; N1-MD, 2 and 6; MD-N2, 3 and 6; NS-N1-MD, 1 and 6; NS-N1-MD-N2, 1 and 7; N1*-MD-N2, 7 and 11. The PCR products were cloned into pET-21d at the NcoI and BamHI or EcoRI sites.

**Table 2:** Primers Used for PCR Cloning *isdB* and *isdC* Fragments.

No.	primer	DNA sequences of primers (5'-3')
1	NS forward	TACCATGGAAGCAGCAGCTGAAGAAACA
2	N1 forward	ACCATGGGCGCACCAAACTCTCGTC
3	MD forward	TACCATGGAAGATTATAAAGCTGAAAA
4	N2 forward	TACCATGGCAAATGAAAAAATGACTGAT
5	N1 reverse	TGGATCCTTATTCAGTTTTGAATTTATCTGC
6	MD reverse	TGGATCCTTATGTTGGTTGTACATTTTG
7	N2 reverse	TCGAATTCTTAATTGGCTTTTGTAAATGC
8	N2 reverse for fusion	GCGGATCCTTTTTCATTTGTTGGTTGTAC
9	N^IsdC^ forward	GCGGATCCGATAGCGGTACTTTGAATTATG
10	N^IsdC^ reverse	TGCTCGAGTTATGTTTGTGGATTTTCTACTTTGTC
11	N1* forward	ACCATGGTGAATCAGGAACTTAGAGAAG

A chimeric gene containing the NS-N1-MD fragment of IsdB and the NEAT domain of IsdC (N^IsdC^) was constructed by PCR cloning. NS-N1-MD was amplified using primers 1 and 8, and cloned into pET-21d at the NcoI and BamHI sites. N^IsdC^ was amplified using primers 9 and 10 and pIDSC [16] as template and fused to the 3′ end of the cloned NS-N1-MD fragment at the BamHI and XhoI sites. The fusion protein (NS-N1-MD-N^IsdC^) produced from this construct contained amino acids Ala40 to Lys341 of IsdB and amino acids 30 to 192 of IsdC. All the clones had no spurious mutations according to DNA sequencing.

### Protein Purification

Recombinant IsdB fragments and NS-N1-MD-N^IsdC^ proteins were expressed in *Escherichia coli* BL21 containing the corresponding plasmid. Bacteria were grown at 37°C in 6 liters of Luria-Bertani broth supplemented with 80 mg/liter ampicillin to optical density at 600 nm of 1.0, and 0.4 mM isopropyl β-D-thiogalactopyronoside was then added to induce protein expression for 8 more hours. All solutions in protein purification were buffered with 20 mM Tris-HCl, pH 8.0, unless specified otherwise. Each bacterial pellet was suspended in 80 ml Tris-HCl, sonicated on ice for 20 min, and the sample was centrifuged at 20,000 g for 15 min to remove cell debris and collect the lysate. All lysates were dialyzed against 5 liters of 20 mM Tris-HCl for 4 h prior to purification.

N1 was purified from inclusion bodies. N1 inclusion bodies were dissolved in 8 M urea, and the denatured N1 was loaded onto a SP sepharose (1.5×5 cm) that was pre-equilibrated with 6 M urea. The column was washed with 30 ml 6 M urea and eluted with a 60 ml gradient of 0 to 100 mM NaCl in 6 M urea. Fractions containing N1 with >90% purity were pooled, and the pooled sample was dialyzed against 20 mM Tris-HCl.

MD lysate was loaded onto a SP sepharose column (2.5×5 cm). The column was washed with 200 ml 20 mM Tris-HCl and eluted with a 100 ml gradient of 0 to 0.2 M NaCl. Fractions containing MD were pooled, and (NH_4_)_2_SO_4_ was added into the sample to 2 M. The sample was loaded onto a phenyl sepharose column (2.5×2.5 cm). The column was washed with 100 ml of 2 M (NH_4_)_2_SO_4_ and eluted with a 50 ml gradient of 2 to 0 M (NH_4_)_2_SO_4_. Fractions containing MD with >80% purity were pooled and dialyzed against 20 mM Tri-HCl buffer.

N2 lysate was loaded onto a SP sepharose column (2.5×10 cm), washed with 20 mM Tris HCl, and the flow through was collected. The flow through was then loaded onto a DEAE sepharose column and eluted with a 120 ml gradient of 0 to 0.2 M NaCl. Fractions containing N2 were pooled and dialyzed against 20 mM Tri-HCl buffer.

NS-N1 lysate was loaded onto a SP sepharose column (2.5×5 cm). The column was washed with 150 ml 20 mM Tris-HCl and eluted with a 200 ml gradient of 0-75 mM NaCl and 40 ml 75 mM NaCl. Fractions containing NS-N1 were pooled, and (NH_4_)_2_SO_4_ was added to the pool to 1.5 M. The sample was loaded onto a phenyl sepharose column (1.0×5 cm). The column was washed with 60 ml 1.5 M (NH_4_)_2_SO_4_ and eluted with a 120 ml gradient of 1.5 to 0.5 M (NH_4_)_2_SO_4_. Fractions containing NS-N1 with >90% purity were pooled and dialyzed against 20 mM Tri-HCl buffer.

MD-N2 was expressed as a mixture of heme-binding form (holo-MD-N2) and heme-free form (apo-MD-N2). MD-N2 lysate was loaded onto a DEAE sepharose column (1.5×7 cm) and washed with 20 ml 20 mM Tris-HCl to collect the flowthrough. The flowthrough was loaded onto a SP sepharose column (2.5×8 cm), and the column was washed with 100 ml Tris-HCl and eluted with a 100 ml gradient of 0 to 100 mM NaCl. Fractions containing apo-MD-N2 were pooled, and the pool was adjusted to 2 M (NH_4_)_2_SO_4_ and loaded onto a phenyl sepharose column (1.5×7 cm). The column was washed with 50 ml 2 M (NH_4_)_2_SO_4_ and then eluted with a 100 ml gradient of 2 to 1 M (NH_4_)_2_SO_4_. The fractions containing apo-MD-N2 with >90% purity were pooled and dialyzed against 20 mM Tri-HCl buffer.

NS-N1-MD lysate was dialyzed against 3 liters of 10 mM Tris HCl, pH 7.0, for 4 h and then loaded onto a SP sepharose column (2.5×5 cm). The column was washed with 100 ml 10 mM Tris HCl, pH 7.0, and then eluted with a 120 ml gradient of 0 to 0.2 M NaCl in Tris-HCl, pH 7.0. Peak fractions containing NS-N1-MD were pooled and dialyzed overnight in 20 mM Tris HCl, pH 8.0. The dialyzed sample was loaded onto a DEAE sepharose column (2.5×5 cm). The column was washed with 20 mM Tris HCl, and the protein was eluted with a 60 ml gradient of 0 to 0.04 M NaCl. The flowthrough and peak fractions were pooled, adjusted to 1.5 M (NH_4_)_2_SO_4_ and loaded onto to a phenyl sepharose column (1×5 cm). The column was eluted with a 50 ml gradient of 1.5 to 0.5 M (NH_4_)_2_SO_4_. Fractions containing NS-N1-MD with >90% purity were pooled and dialyzed against 20 mM Tri-HCl buffer.

N1*-MD-N2 lysate was loaded on a DEAE sepharose column (2.5×7 cm), and the column was washed with 100 ml 20 mM Tris-HCl, pH 8.0, and eluted with a 100 ml gradient of 0 to 0.2 M NaCl. Fractions containing apo-N1*-MD-N2 with >90% purity were pooled and dialyzed against 20 mM Tris-HCl buffer.

NS-N1-MD-N^IsdC^ lysate was loaded on a DEAE sepharose column (2.5×7 cm), and the column was washed with 5 mM Tris-HCl, pH 8.0, to elute out the apo-NS-N1-MD-N^IsdC^. Fractions containing the fusion protein were pooled, and (NH_4_)_2_SO_4_ was added into the pool to 2.0 M. The sample was loaded onto a phenyl sepharose column (1×5 cm), and the column was washed with 30 ml 2 M (NH_4_)_2_SO_4_ and eluted with a 100 ml gradient of 2 to 0 M (NH_4_)_2_SO_4_. Fractions containing apo-NS-N1-MD-N^IsdC^ with >90% purity were pooled and dialyzed against 20 mM Tri-HCl buffer.

### Preparation of Holo-N2

N2 was purified in its heme-free form (apo-N2). Its heme-binding form (holo-N2) was reconstituted from apo-N2 with hemin as described [17]. Briefly, apo-N2 was incubated with excess hemin for 15 min, and holo-N2 was separated from free hemin using a G-25 Sephadex column (1×30 cm).

### Determination of Protein Concentration and Heme Contents

Protein concentrations were determined using a modified Lowry protein assay kit with bovine serum albumin as a standard (Pierce). Heme contents of holo-IsdB proteins and metHb were measured with the pyridine hemochrome assay (ε_418_ = 191.5 mM^−1^ cm^−1^) [32].

### Measurement of Passive Heme Dissociation Using Apo-H64Y/V68F Myoglobin

Passive heme dissociation from holo-N2 or metHb in the absence or presence of MD or NS-N1-MD was measured using H64Y/V68F apomyoglobin as a heme scavenger [30]. Absorbance readings at 600 nm (A_600nm_) was measured as a function of time following the mixing of 2 µM holo-N2 or metHb, MD or NS-N1-MD at indicated concentrations, and 40 µM apo-H64Y/V68F myoglobin. Time-dependent changes in absorption (ΔA_600_) were used to assess the kinetics of heme dissociation reactions.

### Kinetics of Heme Transfer

The rate of fast heme transfers from metHb to IsdB protein constructs and from holo-N2 to apo-IsdC were measured using a stopped-flow spectrophotometer equipped with a photodiode array detector (SX20, Applied Photophysics), as described previously [17]. Briefly, metHb or holo-N2 in one syringe was mixed with apo-protein in another syringe at >5× holo-protein concentrations. Spectra spanning the absorption wavelengths of 250 nm to 700 nm were recorded as a function of time for each reaction.

Rates of slower heme transfers from metHb to IsdB protein constructs or NS-N1-MD-N^IsdC^ were measured by monitoring absorbance changes using a conventional spectrophotometer (SPECTRA^max^ 384 Plus, Molecular Devices). Each holo-protein was incubated with >5× apo-protein concentrations, and absorbance changes at indicated wavelengths were monitored for up to 6 h.

Time courses of absorbance changes obtained from these reactions were fit to a single- or double-phase exponential expression using GraphPad Prism software (GraphPad) to obtain apparent rate constants for heme transfer reactions.

### Estimation of Transferred Heme in Heme Transfer Reactions

Absorption spectra of metHb, apo-IsdB protein constructs or NS-N1-MD-N^IsdC^, and their mixtures were recorded before mixing or at 30 min for the fast metHb/IsdB, metHb/NS-N1-MD-N2, and metHb/N2/NS-N1-MD reactions and at 12 h for the other slower reactions. All the reactions had metHb only as controls at the same time points, and the loss of metHb due to natural dissociation of heme from metHb was not obvious unless a heme scavenger was present. Amounts of transferred metHb heme were estimated using extinction coefficients at 406 nm of 1.7×10^5^, 1.15×10^5^, and 1.1×10^5^ M^−1^·cm^−1^ for metHb-, N2-, and N^IsdC^-bound heme, respectively.
